# Cardiorespiratory fitness does not predict longitudinal changes in manual dexterity, cognition or corticospinal excitability in multiple sclerosis

**DOI:** 10.3389/fnagi.2025.1628832

**Published:** 2026-01-14

**Authors:** Basel Mohamed, Sarah Duraid, Nick W. Bray, Arthur R. Chaves, Michelle Ploughman

**Affiliations:** Recovery and Performance Laboratory, Faculty of Medicine, Memorial University of Newfoundland, St. John’s, NL, Canada

**Keywords:** neurodegeneration, cardiorespiratory fitness, manual hand dexterity, global cognition, transcranial magnetic stimulation, neurological disorder, nine-hole peg test, montreal cognitive assessment

## Abstract

**Introduction:**

Neurodegeneration in multiple sclerosis leads to progressive cognitive and motor impairments. Cardiorespiratory fitness (CRF) is thought to protect against such decline, but its longitudinal effects remain unclear. This study examined whether CRF predicts changes in behavioral (i.e., hand dexterity and cognition) and neurophysiological (i.e., corticospinal excitability, an indicator of corticospinal tract function) outcomes in multiple sclerosis over 2 years, with a focus on participants who experienced no relapses between visits and were, therefore, classified as progression independent of relapse activity (PIRA). We hypothesized that higher baseline CRF would be associated with better follow-up outcomes.

**Methods:**

Participants underwent assessments at two time points (∼2 years apart). CRF was measured using a graded maximal exercise test ($\dot{V}$O_2_max). Behavioral outcomes included the Nine-Hole Peg Test and Montreal Cognitive Assessment. Corticospinal excitability was assessed via transcranial magnetic stimulation of the first dorsal interosseous muscle. Hierarchical regression analyses examined whether baseline CRF predicted change in follow-up scores, controlling for age, sex, and baseline performance.

**Results:**

Among 38 participants (71% female), CRF at baseline did not significantly predict changes in behavioral or physiological outcomes (*p* = 0.178–0.655). Instead, baseline performance was the strongest predictor of follow-up scores. Exploratory analyses revealed inter-individual variability, with some participants improving, declining, or remaining stable over the 2 years. Significant improvements were observed in the Montreal Cognitive Assessment (*p* = 0.002) and non-dominant hand Nine-Hole Peg Test (*p* = 0.036).

**Discussion:**

CRF did not predict longitudinal changes in manual dexterity, cognition or corticospinal excitability in individuals living with multiple sclerosis. Instead, initial performance was the primary determinant of follow-up outcomes, suggesting that achieving better function at baseline (earlier in the disease) is an important rehabilitation target. Variability in longitudinal change underscores the heterogeneous nature of disease progression/improvement and the need for specific, targeted interventions and personalized strategies to disease management.

## Introduction

Neurodegeneration is described as progressive neurological dysfunction and loss of neurons; it is a hallmark of aging and neurological disorders, such as Alzheimer’s disease, multiple sclerosis (MS), and Parkinson’s (Mayne et al., 2020). Neurodegeneration is a complex process, as the exact cause is not entirely understood, and rates vary within and between diseases/disorders (Przedborski et al., 2003). While neurodegeneration is a common hallmark, the resulting physiological and functional consequences differ markedly between diseases, reflecting variation in both rate and severity of decline (Ahmed et al., 2016). For example, neurodegeneration in the peripheral nervous system leads to unstable neuromuscular transmission, resulting in impaired or inconsistent function of the neuromuscular junction and reduced reliability in muscle fiber activation (Hepple and Rice, 2016), while in the central nervous system, it can cause hyperexcitability, characterized by increased spontaneous firing and diminished inhibitory control within motor pathways (Bouteloup et al., 2009). Additionally, neurodegeneration in the hippocampus and motor cortex has been associated with reduced long-term memory (Roux et al., 2021) and slower reaction time (Keleman et al., 2020), respectively. Importantly, cognitive and motor functions, such as working memory, thinking skills, walking, and grasping, arguably represent the most prominent and patient-centered behavioral outcomes in neurodegeneration (Pasko et al., 2022). Exploring such impacted behavioral outcomes in conjunction with underlying physiological mechanisms will promote understanding and, subsequently, refine intervention strategies to help slow neurodegeneration.

Cardiorespiratory fitness (CRF) is associated with a plethora of health benefits, including but not limited to improved heart and respiratory function, enhanced cognitive performance and mental health, and the delayed onset of several chronic diseases, such as osteoarthritis and Type 2 Diabetes (Ruegsegger and Booth, 2018). Previous work has explored CRF as a protective factor in neurodegenerative models. More specifically, studies suggest that maintaining CRF can slow the decline in total gray matter, protect hippocampal subfields, and improve functional connectivity in neurodegeneration (Ahlskog et al., 2011; Bonanni et al., 2022; Bray et al., 2023a; Dougherty et al., 2021). Cross-sectional studies in MS further suggest that higher CRF levels are associated with improved functional outcomes and favorable Transcranial Magnetic Stimulation (TMS) measures (Chaves et al., 2020; Madsen et al., 2019; Zhu et al., 2014). However, other research has shown CRF to be associated with neuropsychiatric burden and have no benefits to cognition in people suffering from neurodegeneration (Ciria et al., 2023; Forbes et al., 2015; Steinberg et al., 2009). Regular participation in physical exercise, which can improve CRF, can facilitate motor performance, particularly in upper extremity tasks for older adults (Hübner and Voelcker-Rehage, 2017; Ploughman et al., 2015). Conversely, other studies report inconsistent changes in motor outcomes with exercise (Bray et al., 2020). Concurrently examining the degree to which CRF may influence physiological processes and behavioral outcomes in neurodegeneration could provide valuable insight into dose-dependent prescriptions, a current goal of exercise physiology research (Barha et al., 2022).

Several cross-sectional studies have investigated the cognitive and motor consequences of CRF in people suffering from neurodegeneration, but we are unaware of any longitudinal work that has done so, with the exception of post and follow-up assessments following an exercise intervention (Chaves et al., 2020). As a result, no study has also looked to understand the underlying physiological changes that may occur concurrently. Therefore, we aimed to investigate whether CRF predicts longitudinal change in behavioral (manual dexterity and cognition) and neurophysiological (corticospinal excitability measured using TMS) outcomes in individuals experiencing neurodegeneration, specifically persons having stable multiple sclerosis (MS). We hypothesized that participants with higher CRF at the first time point would have better physiological and behavioral outcomes at the second, 2 years later.

## Materials and methods

### Design and overview

**FIGURE 1:**
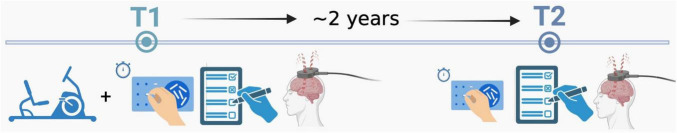
Schematic representation of study outcomes and data collection timeline. Study outcomes include cardiorespiratory fitness via a $\dot{V}$O_2_max test, manual hand dexterity via the Nine-hole Peg Test, global cognition via the Montreal Cognitive Assessment, and corticospinal tract function via transcranial magnetic stimulation. With the exception of cardiorespiratory fitness, all outcomes were reassessed 2 years later at T2 (i.e., follow-up). T1, baseline.

We conducted a longitudinal study with time points (i.e., T1 and T2) ∼2 years apart; we used a convenience sample from the Health Research Innovation Team in Multiple Sclerosis (HITMS) project, which is currently collecting data on disease symptoms, neuro-immune and blood profiles, and cognitive and physical function in persons living with MS in Newfoundland and Labrador, Canada (Fifield et al., 2025). To be included in the present study, participants had to: (1) be at least 18 years of age; (2) satisfy the 2010/2017 McDonald criteria for MS (Polman et al., 2011; Thompson et al., 2018); (3) complete CRF testing at T1; (4) complete behavioral (i.e., Nine-Hole Peg test (9HPT), Montreal Cognitive Assessment (MoCA), and neurophysiological outcomes (i.e., TMS) at T1 and T2; and (5) have no documented relapse in the 3 months prior to T1 and during the interval between T1 and T2. Participants who experienced a relapse during the study window were excluded because acute relapses can cause temporary or fluctuating changes in cognitive, motor, and neurophysiological function, likely confounding the interpretation of longitudinal changes (Benedict et al., 2021; Nickerson et al., 2015). Assessments took ∼3 h in a neurorehabilitation research laboratory (Figure 1). We collected demographic data through a combination of health records and in-person assessments. Participants provided written informed consent per the Declaration of Helsinki, and the local Human Research Ethics Board approved the study (HREB Ref: 2015.103).

### Assessments

#### Fitness testing

We determined CRF via a graded exercise test and calorimetry measuring maximal oxygen uptake (i.e., $\dot{V}$O_2_max). Participants performed the graded exercise test on a total body recumbent stepper (NuStep, Ann Arbor, Michigan) as it is a safe, feasible, and valid exercise test for persons living with MS (Billinger et al., 2008). The graded exercise test followed a previously published protocol from our group (Anvar et al., 2024; Kelly et al., 2017). Before beginning, we recorded the heart rate and blood pressure to check for possible contraindications. These vitals were then monitored intermittently throughout the graded exercise test and again during the cool-down. After confirming no contraindications existed, participants completed a 2-min warmup at resistance level 1/10 on the NuStep’s manufacturer-provided 1–10 resistance scale, where 1 is minimal resistance and 10 is maximal resistance. The NuStep 1–10 scale corresponds to a power output range of approximately 0–800 watts. Following the warm-up, participants maintained a cadence of 80 strides per minute, but we increased the resistance level to 3/10—this signified the start of the test. Every 2 min, we increased the resistance by one unit until the participant reached level 10 (maximal NuStep resistance), succumbed to exhaustion, or if the supervising exercise physiologist identified clinical indications for discontinuation (e.g., abnormal blood pressure response, excessive fatigue, dizziness, etc.). If a participant reached level 10 without exhaustion, we augmented the workload by increasing strides per minute by 10 units every 2 min until exhaustion. Upon test completion, participants performed a 2-min cool-down similar to the warm-up protocol, followed by a revaluation of blood pressure and heart rate. From calorimetry (Moxus; AEI Technologies, Kempele, Finland), we obtained the highest or the maximum relative rate of oxygen consumption ($\dot{V}$O_2_max). Achievement of $\dot{V}$O_2_max was determined based on attainment of at least two of the following criteria: (i) a plateau in oxygen uptake (<80 mL ⋅ min^−1^) despite increasing workload; (ii) a respiratory exchange ratio ($\dot{V}$CO_2_/$\dot{V}$O_2_) ≥ 1.10; and/or (iii) peak heart rate within ± 10 bpm of the age-predicted maximum, calculated as 206.9–(0.67 × age) or 164–(0.7 × age) if prescribed beta-blockers (Ferguson, 2014). We refer to each individual’s highest achieved oxygen consumption as $\dot{V}$O_2_max throughout this manuscript, as it reflects their maximal attainable effort relative to their MS-induced physiological and neurological constraints. This approach aligns with prior literature using similar clinical populations and protocols (Langeskov-Christensen et al., 2014; Poole and Jones, 2017).

#### Nine-Hole Peg Test

We used the Nine-Hole Peg Test (9HPT) to assess manual hand dexterity (Oxford Grice et al., 2003). Using only one hand and doing so as quickly as possible, participants removed wooden pegs, one-by-one, from a container and placed them into one of 9-holes on a board (7 mm diameter, 32 mm length). Once all pegs were inserted, participants moved the pegs back to the original container one-by-one. We had participants complete the test two times per hand and then averaged completion time to produce a final score for both the dominant and non-dominant sides in seconds. We randomized the testing order (i.e., dominant versus non-dominant) within and between participants to minimize a practice effect (Feys et al., 2017).

#### Montreal Cognitive Assessment

To assess cognitive function, we had participants complete the Montreal Cognitive Assessment (MoCA) (Nasreddine et al., 2005). Briefly, the MoCA is a 10-min test that identifies cognitive impairment by assessing global cognition via domain-specific tasks, such as attention, concentration, executive function, memory, language, visuoconstructional skills, conceptual thinking, calculations, and orientation. All assessors completed MoCA certification prior to administering any tests (Rosca et al., 2020).

#### Transcranial magnetic stimulation

Similar to our recent work, we used single-pulse TMS to assess corticospinal excitability (Chaves et al., 2021; MacKenzie et al., 2025). In brief, we delivered monophasic pulses using a BiStim 2002 (Magstim Co., Whitland, United Kingdom) stimulator with a 70 mm figure-of-eight coil to elicit motor evoked potentials from the first dorsal interosseous muscle of both hands. To measure electromyography activity and motor evoked potentials of the hands, we first cleaned the skin surface before placing foam surface electrodes (Kendall 200 Covidien, Mansfield, MA) over the belly of the first dorsal interosseous muscle (active electrode), the ulnar styloid process (ground electrode) and the interphalangeal joint of the index finger (reference electrode). Electromyography activity was transmitted to a recording system (Brainsight*™*, Rogue Research, Montreal, QC, Canada; 3 kHz sampling, 2,500 V/V amplification, 600 V/V gain, bandwidth of 16–550 Hz) and analyzed using Signal Software version 6.06 (Cambridge Electronic Design Ltd., Cambridge, United Kingdom).

We used Brainsight*™* neuronavigation to ensure precise TMS coil placement and orientation over the scalp and to determine the motor “hotspot,” defined as the motor cortex area that elicits the largest peak-peak motor evoked potential amplitude (μV) in the target muscle (i.e., first dorsal interosseous muscle) (Rossini et al., 2015). With the participant seated, we administered monophasic posterior-anterior pulses, with the coil positioned tangentially to the scalp and oriented posterolaterally at a 45° angle to the midsagittal line. We delivered supra-threshold stimulations at different sites over the primary motor cortex to identify the motor hotspot.

Using the identified motor hotspots, we measured the active motor thresholds (AMT) for both hemispheres, but our analyses emphasized the weaker hand, as determined using a calibrated pinch gauge (B&L Engineering, Santa Ana, CA, United States). We focused on the weaker hand because in people living with MS, the corresponding hemisphere demonstrates lower excitability and higher inhibition, suggesting a greater potential for neuroplastic changes (Garvey et al., 2001; Lepley et al., 2020). Further, measures from the weaker side yield stronger predictions of clinical outcomes, such as hand dexterity, walking performance, cognitive speed, fatigue, disability, and heat sensitivity (Garvey et al., 2001; Groppa et al., 2012; Rossini et al., 2015; Snow et al., 2019). AMT was defined as the minimum TMS intensity required to elicit at least 5/10 motor-evoked potentials with amplitudes ≥ 200 μV during slight tonic contraction of the muscle. This contraction was standardized at ∼10% of each participant’s maximum pinch grip force, which we determined by asking participants to hold a calibrated pinch gauge between the pads of their thumb and index finger and then perform a maximal voluntary contraction (i.e., pinch as hard and as fast as possible). Higher AMT values (higher threshold and less excitability) may indicate demyelination and axonal damage (Snow et al., 2019). From the AMT, we determined the cortical silent period (CSP; ms) or the interruption of electromyography activity following suprathreshold TMS stimulation, as per the protocol described by Chaves et al. (2021). The length of the CSP indicates an inhibitory refractory period that represents GABA-mediated inhibition of the corticospinal tract (Ahern et al., 2023; Connors et al., 1988; Mehta et al., 2021). Longer CSP has been associated with lower fitness and weakened capacity for long-term potentiation in people with MS (Chaves et al., 2019a). In addition to absolute AMT values, we calculated an AMT asymmetry ratio by dividing the AMT of the weaker hemisphere by that of the stronger hemisphere, providing a relative measure of interhemispheric excitability imbalance (Chaves et al., 2019b; Chaves et al., 2021). Our previous work suggests that imbalance of excitability between the hemispheres is suggestive of neurodegeneration (Chaves et al., 2021). In summary, the primary TMS outcome measures were: (1) AMT; (2) CSP; and (3) AMT asymmetry ratio.

### Statistical analysis

We conducted a hierarchical regression analysis to determine predictors of our dependent variables (i.e., 9HPT, MoCA, and TMS outcomes (i.e., AMT, CSP, asymmetry ratios) at T2. Model 1 controlled for age, sex, days between visits, and the dependent variable (i.e., 9HPT, MoCA, etc.) score at T1, while Model 2 added $\dot{V}$O_2_max at T1 to determine the influence of CRF at T1 on the dependent variable at T2. All outputs from these analyses satisfied the required assumptions. We removed unusual (i.e., outliers, influential points, etc.) points unique to each analysis. All outputs satisfied the assumptions of hierarchical regression, including “unusual data points” or those with studentized deleted residuals values > ± 3 (i.e., outliers) and leverage points > 0.2 or influential points via Cook’s distance > 1; data points flagged as unusual were removed and excluded from their respective analysis. To better understand the baseline relationship between variables, we performed a correlation analysis between CRF and all other variables (i.e., age, MoCA, 9HPT, AMT, AMT asymmetry, CSP) using a Spearman correlation test; Spearman was preferred for our relatively smaller (*n* < 50) sample size. In addition, because of the heterogeneity of our sample, we performed a sensitivity analysis, stratifying the sample by age (median split: ≤ 50 vs. > 50 years) and Expanded Disability Status Scale (EDSS) score (<1 vs. ≥ 1). The EDSS is a standardized clinical scale that quantifies MS disability, ranging from 0 (normal neurological examination) to 10 (death due to MS) (Kurtzke, 1983). In the MS literature, stratification is commonly done either at 40 years or by a median split (Jones and Amtmann, 2015; Lefort et al., 2022). Because relatively few of our participants were under 40, we opted for a median-based split to maintain relatively balanced subgroup sizes. Classification was based on a threshold of 5 change from their T1 value. As a post hoc exploratory analysis, we categorized participants as “improved,” “declined,” or “no change” in functional outcomes over the 2-year period using a 5% threshold relative to their T1 value; changes greater than ± 5% indicated improvement or decline (depending on direction), while changes ≤ 5% indicated no change. We adopted this 5% change threshold as a practical variation of the Minimal Clinically Important Difference (MCID). Minimal clinically important difference provides a useful framework for interpreting the clinical relevance of change, but there is currently no consensus on a single minimal clinically important difference value for most MS-related outcomes, with studies reporting a wide range of possible thresholds for the same measure. Therefore, in the absence of standardized definitions or an established operational framework (Beaton et al., 2002; McGlothlin and Lewis, 2014; Wells et al., 2001), we selected a 5% threshold to provide a consistent, interpretable, and feasible means of classifying change while maintaining demographic balance and analytic robustness. We performed all statistical analyses using IBM SPSS Statistics, version 29 (IBM Canada Ltd., Markham, Ontario). *p* < 0.05 indicate statistical significance.

## Results

### Demographic characteristics of the participants

**FIGURE 2:**
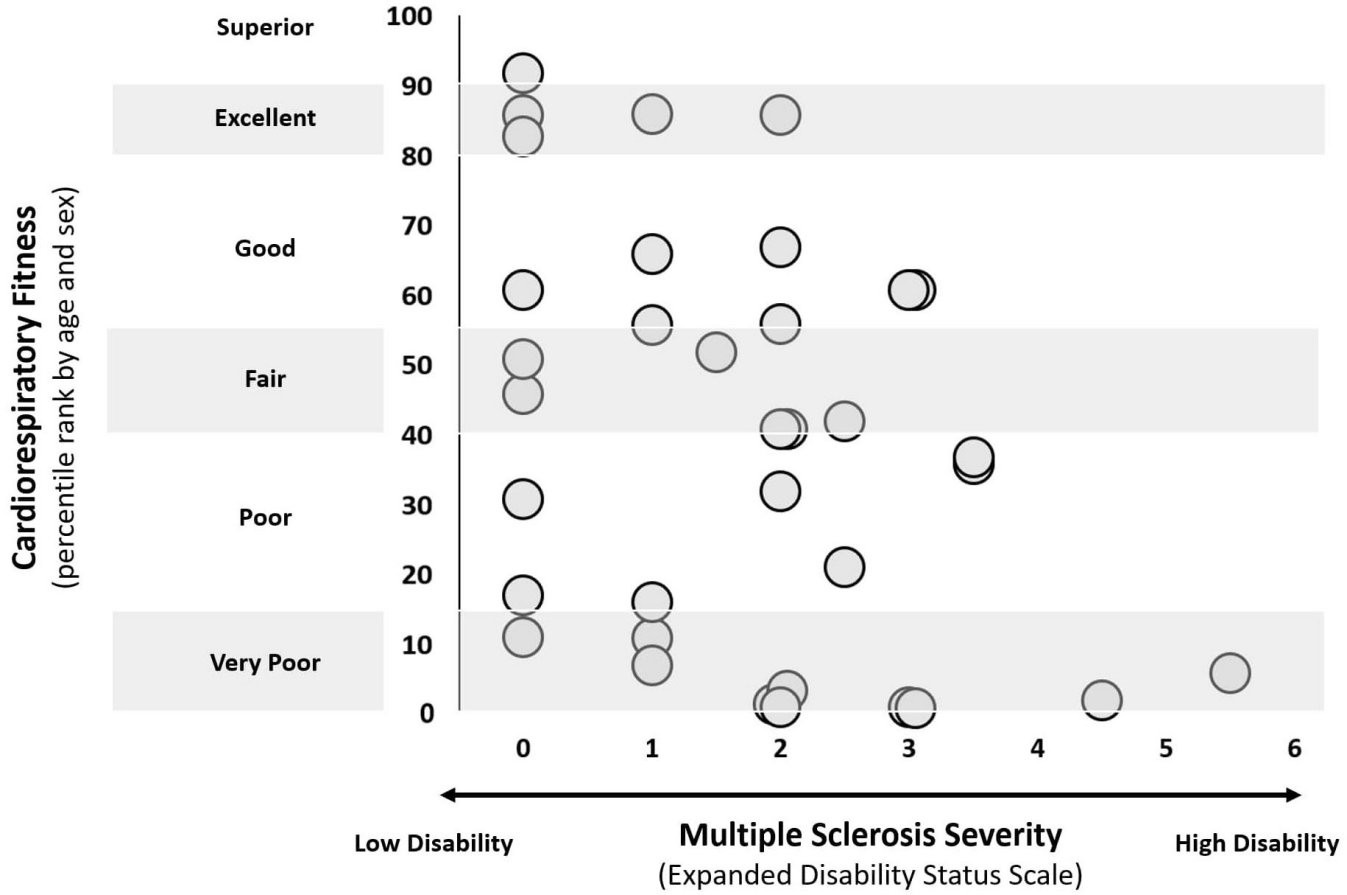
Cardiorespiratory fitness and severity of multiple sclerosis (MS) disability, as measured by the Expanded Disability Status Scale (EDSS). 35 people with MS were ranked according to age- and sex-specific normative percentiles for cardiorespiratory fitness (McGlothlin and Lewis, 2014).

Our sample included 38 participants, of whom 27 were female (Table 1). The average duration of the $\dot{V}$O_2_max test, excluding warmup and cooldown, was 15.4 min (ranging from 6.87 to 22.92 min) with 71% of participants meeting max criteria. With respect to disease-modifying therapy, it is important to note that there is currently no universal consensus on their efficacy classification. However, consistent with prior literature, we categorized them as high- or moderate/low-efficacy treatments (Freeman et al., 2022; Harding et al., 2019). As such, one, 26, and 11 participant(s) was/were receiving high-efficacy, moderate/low-efficacy, and no disease-modifying therapies, respectively. Our sample also demonstrated heterogenous cardiorespiratory fitness among participants with EDSS scores of 0–2, alongside a general trend of decreasing fitness with increasing (i.e., worse) EDSS scores in those with EDSS scores > 2 (Figure 2). We identified and subsequently removed three participants (*n* = 35) from the non-dominant hand 9HPT analysis as their performance was flagged as unusual. Additionally, we removed one (*n* = 37) from AMT and 9 (*n* = 29) and 10 (*n* = 28) from CSP and asymmetry ratios, respectively, due to incomplete TMS sessions. At baseline, Spearman correlations showed significant but weak associations with AMT (ρ = –0.340, *p* = 0.040), dominant 9HPT (ρ = –0.336, *p* = 0.039), and CSP (ρ = –0.396, *p* = 0.030); correlations with MoCA, non-dominant 9HPT, and AMT asymmetry did not reach statistical significance (Supplementary Table 1).

**TABLE 1:** Descriptive characteristics of sample (*n* = 38).

Variable	Mean ± SD
Female: male	27:11
Age (years)	48.53 ± 11.53
Height (meters)	1.69 ± 0.08
Weight (kilograms)	79.39 ± 17.69
Expanded Disability Status Scale	1.70 ± 1.40
Duration of $\dot{V}$O_2_max test (minutes)	15.4 ± 3.9
Days between T1 and T2	773.45 ± 303.73
Efficacy of Disease-Modifying Therapy	
High	1
Mod/Low	26
None	11

Values are presented as mean ± standard deviation, except for the number of females and males, and the number of participants categorized by Disease-Modifying Therapy efficacy level. Unless otherwise specified, all values are from the T1 (baseline) timepoint; “Days between T1 and T2” reflects the interval between baseline and follow-up. VO_2_max test duration is reported in minutes (with decimal fractions). T1, baseline; T2, follow-up.

### T1 cardiorespiratory fitness as a predictor of T2 outcome variables

After controlling for age, sex, time between visits, and the outcome-specific T1 score (i.e., T1 MoCA in the model exploring T2 MoCA), the strongest predictor of each T2 outcome was the T1 measure. Therefore, $\dot{V}$O_2_max at T1 did not significantly predict dominant (β = -0.095, *p* = 0.178) or non-dominant (β = -0.051, *p* = 0.241) 9HPT score at T2 (Table 2). Similarly, $\dot{V}$O_2_max at T1 was not a significant predictor of MoCA (β = -0.016, *p* = 0.655) or any TMS outcomes at T2: AMT (β = 0.120, *p* = 0.450); CSP (β = -0.451, *p* = 0.589); and asymmetry ratios (β = 0.004, *p* = 0.425). The sensitivity analyses yielded results consistent with the primary analysis; therefore, there was no evidence of differential predictive effects across age and EDSS subgroups (Supplementary Tables 2a–5b).

**TABLE 2a:** Hierarchical regression for hand dexterity (Nine-Hole Peg Test; 9HPT) and global cognition (Montreal Cognitive Assessment; MoCA).

Variables	*R* ^2^	F change	B (SE)	95% CI for B		β	*p*
				LL	UL		
**Hand dexterity (9HPT)**							
Dominant hand (*n* = 38)							
Model 1	0.713	20.483					*** < 0.001***
*Age*			–0.033 (0.040)	–0.114	0.048	–0.077	0.414
*Sex*			0.898 (0.988)	–1.113	2.909	0.085	0.370
*Days between T1 and T2*			0.001(0.002)	–0.002	0.004	0.050	0.595
*9HPT at T1*			0.961 (0.111)	0.736	1.186	0.811	*** < 0.001***
Model 2	0.729	1.897					***< 0.001***
*Age*			–0.049 (0.041)	–0.133	0.034	–0.110	0.239
*Sex*			1.374 (1.035)	–0.733	3.482	0.122	0.194
*Days between T1 and T2*			4.426E-4 (0.002)	–0.003	0.004	0.027	0.772
*9HPT at T1*			0.919 (0.113)	0.688	1.150	0.746	*** < 0.001***
$\dot{V}$O_2_max *at T1*			–0.095 (0.069)	–0.235	–0.045	–0.127	0.178
Non-dominant hand (*n* = 35)							
Model 1	0.931	100.672					*** < 0.001***
*Age*			0.011 (0.025)	–0.040	0.061	0.021	0.665
*Sex*			0.396 (0.599)	–0.826	1.619	0.032	0.513
*Days between T1 & T2*			0.002 (0.001)	0.000	0.004	0.078	0.117
*9HPT at T1*			1.057 (0.054)	0.947	1.166	0.947	*** < 0.001***
Model 2	0.003	1.435					*** < 0.001***
*Age*			3.96E-4 (0.026)	–0.054	0.053	–0.01	0.988
*Sex*			0.659 (0.634)	–0.637	1.955	0.050	0.307
*Days between T1 and T2*			0.001 (0.001)	–0.001	0.003	0.055	0.261
*9HPT at T1*			1.035 (0.056)	0.920	1.150	0.879	*** < 0.001***
$\dot{V}$O_2_max *at T1*			–0.051 (0.043)	–0.139	0.036	–0.057	0.241
**Global cognition (MoCA) (*n* = 38)**							
Model 1	0.542	9.765					*** < 0.001***
*Age*			–0.022 (0.021)	–0.064	0.020	–0.127	0.288
*Sex*			0.282 (0.503)	–0.741	1.305	0.066	0.579
*Days between T1 and T2*			0.001 (0.001)	–0.001	0.003	0.152	0.206
*MoCA at T1*			0.579 (0.106)	0.364	0.794	0.646	*** < 0.001***
Model 2	0.545	0.203					*** < 0.001***
*Age*			–0.025 (0.022)	–0.070	0.019	–0.137	0.259
*Sex*			0.357 (0.536)	–0.734	1.448	0.080	0.510
*Days between T1 and T2*			0.001 (0.001)	–0.001	0.003	0.136	0.262
*MoCA at T1*			0.584 (0.107)	0.365	0.803	0.648	*** < 0.001***
$\dot{V}$O_2_max *at T1*			–0.016 (0.035)	–0.087	0.055	–0.054	0.655

**TABLE 2b:** Hierarchical regression for corticospinal excitability, measured using transcranial magnetic stimulation.

Variables	*R* ^2^	F change	B (SE)	95% CI for B		β	*p*
				LL	UL		
**Corticospinal excitability (TMS)**							
AMT (*n* = 37)							
Model 1	0.672	16.389					*** < 0.001***
*Age*			–0.127 (0.093)	–0.317	0.063	–0.138	0.182
*Sex*			1.438 (2.294)	–3.235	6.112	0.063	0.535
*Days between T1 and T2*			–0.004 (0.003)	–0.011	0.003	–0.121	0.240
*AMT at T1*			0.821 (0.107)	0.603	1.039	0.778	*** < 0.001***
Model 2	0.678	0.586					*** < 0.001***
*Age*			–0.106 (0.098)	–0.305	0.093	–0.110	0.287
*Sex*			0.988 (2.383)	–3.872	5.849	0.042	0.681
*Days between T1 and T2*			–0.003 (0.004)	–0.011	0.004	-0.097	0.348
*AMT at T1*			0.835 (0.109)	0.612	1.057	0.780	*** < 0.001***
$\dot{V}$O_2_max *at T1*			0.120 (0.156)	–0.199	0.439	0.078	0.450
CSP (*n* = 29)							
Model 1	0.526	6.666					*** < 0.001***
*Age*			0.418 (0.502)	–0.617	1.454	0.117	0.413
*Sex*			21.582 (11.811)	–2.795	45.959	0.257	0.080
*Days between T1 and T2*			–0.043 (0.016)	–0.076	–0.009	–0.364	***0.016***
*CSP at T1*			0.609 (0.145)	0.310	0.908	0.590	*** < 0.001***
Model 2	0.532	0.300					***0.002***
*Age*			0.325 (0.537)	–0.786	1.436	0.086	0.552
*Sex*			23.239 (12.363)	–2.335	48.813	0.268	0.073
*Days between T1 and T2*			–0.044 (0.017)	–0.078	–0.009	–0.371	***0.016***
*CSP at T1*			0.567 (0.166)	0.224	0.909	0.488	**0.002**
$\dot{V}$O_2_max *at T1*			–0.451 (0.822)	–2.151	1.250	-0.078	0.589
Asymmetry ratios (*n* = 28)							
Model 1	0.542	6.818					*** < 0.001***
*Age*			-0.007 (0.004)	–0.014	0.001	–0.269	0.069
*Sex*			0.045 (0.076)	–0.113	0.203	0.083	0.564
*Days between T1 and T2*			0 .000 (0.00)	.000	0.000	–0.087	0.545
*Asymmetry ratios at T1*			0.739 (0.168)	0.391	1.086	0.620	*** < 0.001***
Model 2	0.556	0.455					***0.002***
*Age*			–0.006 (0.004)	–0.014	0.002	–0.227	0.124
*Sex*			0.027 (0.080)	–0.140	0.193	0.047	0.743
*Days between T1 and T2*			0.000 (0.00)	0.000	0.000	–0.052	0.717
*Asymmetry ratios at T1*			0.773 (0.174)	0.411	1.135	0.630	*** < 0.001***
$\dot{V}$O_2_max *at T1*			0.004 (0.005)	-0.007	0.016	0.116	0.425

9HPT, Nine-Hole Peg Test; MoCA, Montreal Cognitive Assessment; AMT, Active Motor Threshold; CSP, Cortical silent Period; T1, Baseline; T2, Follow-up; R^2^, coefficient of determination; F Change = F-statistic for R^2^ change, B = unstandardized regression coefficient; SE, standard Error; CI, Confidence Interval; LL, Lower Limit; UL, upper limit; β = standardized regression coefficient. Bolded and italicized *p*-values indicate statistical significance (i.e., *p* < 0.05).

### Variability in outcome measures over 2 years

**FIGURE 3:**
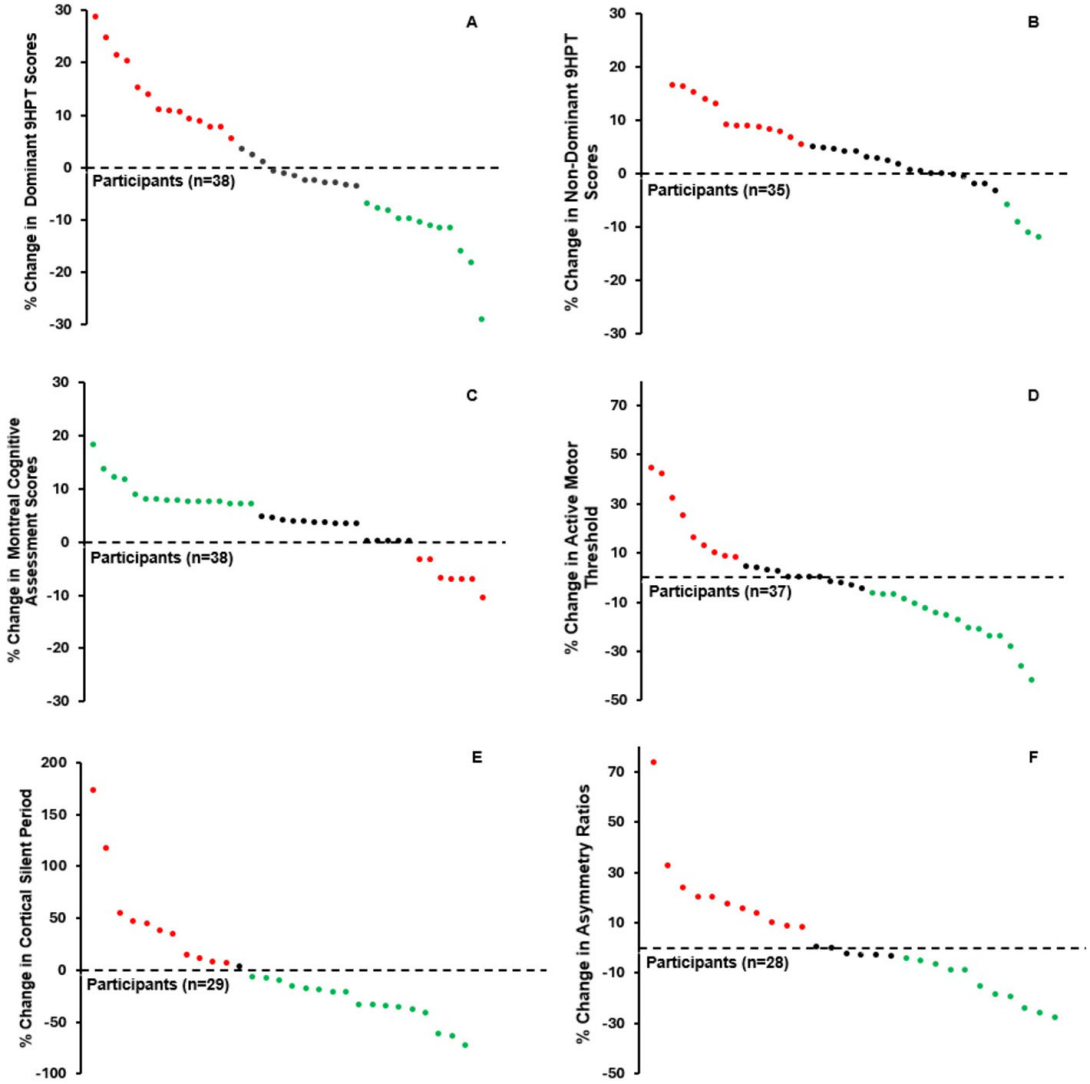
Percentage change in scores from T1 (i.e., baseline) to T2 (i.e., follow-up). Each “dot” represents an individual participant. “Improvement” and “Decline” scores are considered a change =± 5% from T1 and T2, and are represented by green and red dots, respectively. Therefore, an increase in score for one outcome could reflect an “improvement,” while for others, it could reflect a “decline.” Black dots reflect consistent (<5% change between T1 and T2) scores. **(A)** *n* = 38, 9HPT = Nine-Hole Peg Test; **(B)** *n* = 35; **(C)** *n* = 37; **(D)** *n* = 38; **(E)** *n* = 29; **(F)** *n* = 28.

Because our hypothesis was incorrect, we conducted an exploratory, post-priori analysis to better understand our dataset and the degree of change in our outcome measures. Further, we performed a paired *t*-test to compare T1 and T2 for each dependent variable. Change in dominant hand 9HPT scores over 2 years showed that 12 (31.5%) participants decreased (i.e., improved), 14 (37%) increased (i.e., worsened), and 12 (31.5%) remained the same (i.e., within 5% change). Such change variability was echoed through our other outcome variables (Figure 3). Despite the variability, the average change in some variables had statistical significance. Specifically, MoCA (*p* = 0.002) and non-dominant hand 9HPT (*p* = 0.036) showed significant improvement from T1 to T2 (Table 3).

**TABLE 3:** Dependant variable values at T1 and T2.

Variable	T1 (baseline)	T2 (follow-up)	*p*-value
$\dot{V}$O_2_max (mL/kg/min)	25.33 ± 7.90	24.65 ± 9.40	0.700
**9 Hole Peg Test (seconds)**			
Dominant	20.97 ± 4.17	21.20 ± 4.83	0.605
Non-dominant^a^	22.34 ± 5.56	23.27 ± 6.39	***0.036***
Montreal Cognitive Assessment	26.79 ± 2.26	27.68 ± 1.95	***0.002***
**Corticospinal excitability**			
Active motor threshold^b^	35.16 ± 9.88	33.41 ± 8.51	0.180
Cortical silent period^c^	72.12 ± 35.27	64.44 ± 35.57	0.227
Asymmetry ratios^d^	1.045 ± 0.21	1.06 ± 0.24	0.712

All values are mean ± standard deviation. We used a paired samples *t*-test to compare T1 and T2 values. mL/kg/min = milliliters of oxygen consumed in 1 min, per kilogram of body weight.

*^a^n* = 35,

*^b^n* = 37.

*^c^n* = 29,

*^d^n* = 28. Bolded and italicized *p*-values indicate statistical significance (i.e., *p* < 0.05).

## Discussion

We aimed to determine whether CRF protects against longitudinal changes in community-dwelling persons living with stable MS. Indeed, higher levels of CRF and engagement in exercise have been linked to lower relapse risk in MS (Proschinger et al., 2022). Importantly, participants who relapsed just prior to (i.e., < 3-months) or during the study period were excluded from our study/analysis. While this may mean our sample represents a somewhat healthier subgroup of people with MS, this design allowed us to specifically examine progression independent of relapse activity (PIRA) (Tur et al., 2023). Taken together, although this approach may limit the generalizability of our findings to the full spectrum of people with MS, it provides a unique opportunity to isolate neurodegenerative changes occurring outside of relapses, which is increasingly recognized as a primary driver of long-term disability accumulation in people with MS. Within this framework, we show that CRF at T1 (baseline) did not predict T2 (follow-up) scores in any behavioral or neurophysiological variable. Instead, with the exception of the number of days between visits for CSP, the T1 score for each outcome was the sole significant predictor of its corresponding T2 score. Importantly, we also observed considerable variability in change scores between T1 and T2, including increases, decreases, and no change, with the most striking being a significant improvement in non-dominant 9HPT time and MoCA score. Together, these results suggest that T1 performance and not CRF predict change in MS, but that MS is not synonymous with continuous and linear decline in behavioral and neurophysiological function.

CRF is widely regarded as neuroprotective in aging and neurodegeneration due to its positive associations with brain structure and function, enhanced cerebral blood flow, and improved metabolic and inflammatory profiles – all of which contribute to maintaining cognitive and motor function (Blackmore et al., 2009; Honea et al., 2009; Smith et al., 2021). However, in our sample, CRF at T1 did not predict hand dexterity (i.e., dominant or non-dominant 9HPT time), global cognition (i.e., MoCA), or corticospinal excitability (i.e., TMS outcomes of AMT, CSP, and asymmetry ratios). We believed CRF would predict and ultimately protect against neurodegeneration-induced decline in behavioral and neurophysiological variables based upon prior cross-sectional studies in healthy and clinical populations demonstrating CRF to be positively associated with such outcomes (Buragadda and Ploughman, 2025; Hübner and Voelcker-Rehage, 2017; Ploughman et al., 2015; Sandroff et al., 2019; Spirduso et al., 1988); meaning, higher CRF has been typically associated with better performance in hand dexterity, global cognition, and corticospinal excitability. The absence of a relationship between T1 CRF and T2 outcomes may reflect sample heterogeneity; that is, variable disease progression, a common occurrence across individuals suffering from MS, likely diluted any consistent predictive signal of CRF. To this end, and as a reminder, some of our participants demonstrated declines in performance (i.e., > –5% change), while others remained stable (i.e., < –5% change) or even improved (i.e., > +5% change). Importantly, heterogeneity in MS progression is well-documented; while some individuals experience rapid declines, others remain stable (Baecher-Allan et al., 2018; Engelhardt et al., 2022) or, at least in our sample, demonstrate modest but statistically significant improvements, as per non-dominant 9HPT (i.e., hand dexterity) and MoCA (i.e., global cognition) score. It is possible that compensatory neuroplasticity, stable disease pathology, and/or ongoing treatment contributed to the observed improvements (Holm et al., 2022; Robertson and Moreo, 2016). Interestingly, some individuals diagnosed with mild cognitive impairment, a prodromal or transitional stage between “normal” aging and a dementia syndrome, also experience an improvement in cognition, although the underlying mechanisms remain unclear (Canevelli et al., 2016).

Variability in disease progression may be attributed to several complex and interconnected factors. Firstly, disease progression reflects a complex interplay between age, genetics, lifestyle, and environmental exposures, among other factors (Van Hijfte et al., 2022; Zeydan and Kantarci, 2020). In addition to MS, Alzheimer’s, cancer, and HIV/AIDS are just some of the other diseases that demonstrate heterogeneity in progression (Bray et al., 2023b; Jamal-Hanjani et al., 2015; Lukashov and Goudsmit, 1998). Second, MS disease progression is characterized by periods of relapse and remission, when individuals experience a significant worsening or improvement in symptoms, lasting for a few days to several weeks or months (Iuliano et al., 2008). Third, various disease-modifying therapies are approved for treatment in MS (Robertson and Moreo, 2016). Of course, all disease-modifying therapies aim to help with MS symptoms, but none ultimately cure the disease. Further, their effects are not uniform and individuals experience a variety of side effects, even when using the same medication (Robertson and Moreo, 2016). Along these lines, individuals may be engaged in several other pharmacological and non-pharmacological interventions, including newer, more effective therapies, all of which could impact disease progression and symptom management (Torkildsen et al., 2016). Finally, one more factor to consider relative to disease variability is whether our participants truly reached $\dot{V}$O_2_max or were only able to achieve a $\dot{V}$O_2_peak during their CRF testing. $\dot{V}$O_2_peak is the highest recorded oxygen consumption during a test, but it does not necessarily reflect a true physiological maximum (i.e., $\dot{V}$O_2_max); failure to achieve a true $\dot{V}$O_2_max may be attributed to a variety of overlapping factors, one being localized muscle fatigue, which is prominent in MS populations (Bray et al., 2023b; Jamal-Hanjani et al., 2015). Ultimately, our CRF assessments may have captured peak oxygen uptake rather than true $\dot{V}$O_2_max, limiting the accuracy of our fitness measurements and predictive ability for some participants (Jamal-Hanjani et al., 2015). In this regard, it is important to highlight that the heterogeneous nature of MS was also reflected in the wide range of CRF levels observed in our sample. In particular, individuals with lower EDSS scores (< 2) displayed fitness levels ranging from very poor to superior (Ferguson, 2014), while those with EDSS scores > 2 generally exhibited a downward trend in fitness with advancing disability.

A discussion surrounding outcome variability is incomplete without considering participants’ baseline or starting point. We found that every outcome’s T2 score was significantly predicted by their T1 score. Such findings suggest that initial levels of impairment, and not CRF, play a critical role in shaping long-term trajectories; this emphasizes the importance of early identification and intervention in persons living with MS as it provides an opportunity to minimize disease progression and maximize health. For example, it is possible that baseline disability level moderates the extent to which individuals benefit from physical activity; at the very least, there may be an interaction. Specifically, those with lower disability (i.e., better function) may have a more intact neurophysiological system, conferring a greater propensity for adaptive neuroplasticity in response to exercise. At the same time, those with lower disability (i.e., better function) are likely capable of engaging in more difficult or demanding physical exercise, thereby inducing greater physiological changes that translate to reduced or slowed disease progression (Ksiazek-Winiarek et al., 2015; Pilutti et al., 2015). A similar perspective exists in other neurodegenerative diseases. For example, mild cognitive impairment is considered a more optimal period for intervention than later disease stages, such as mild or moderate Alzheimer’s (Albert et al., 2011). Similarly, individuals with pre-frailty have more capacity and can therefore engage in more demanding physical exercise than their counterparts with more severe frailty (Bray et al., 2016). Importantly, the early or transitional stages of MS are an active area of research. For example, our group recently explored the relationship between objective and subjective hand dexterity in people living with mild MS (i.e., score of 3 or less on the EDSS). We found that robotic-based measures of hand dexterity, but not the gold-standard 9HPT, differentiated between those that did and did not report subjective hand impairments (Bray et al., 2024).

Despite our findings and the value added to the literature, our study is not without limitations. The time between the T1 and T2 was approximately 2 years, but this varied between participants, largely due to the disruptions caused by the COVID-19 pandemic. Although we controlled for this variability, such delays introduced temporal confounding, which may have made it harder to track consistent change patterns over time. Further, it is possible that a 2-year follow-up was too short to capture meaningful neurodegenerative change or to observe the protective effects of CRF, particularly in a condition as heterogeneous as MS. Another limitation is the wide age range of participants (i.e., 19–65 years). CRF may have had stronger predictive ability in behavioral and physiological outcomes if we focused exclusively on younger or older adults. Importantly, aging is associated with an increased risk of various other diseases and syndromes, such as pain, hearing loss, and polypharmacy, all of which can individually and synergistically have a negative impact on health (Livingston et al., 2024; Maher et al., 2014; Palmer et al., 2023). Along these lines, we previously demonstrated that individuals with MS performed hopping—a muscle power activity—at a level comparable to people 30 years older but without MS (Kirkland et al., 2022); this highlights the phenomenon of accelerated physiological aging in MS and underscores the complexity of interpreting age-related fitness measures in this population. In regard to both aging and MS, previous work has demonstrated that both experiences are sex-specific; unfortunately, our sample size precluded us from individually analyzing males and females. Additionally, Magnetic Resonance Imaging (MRI) data were not included due to variability in clinical imaging schedules, accessibility, and cost. The absence of MRI-based markers limits our ability to directly relate functional and neurophysiological findings to underlying structural disease activity. Finally, our study was geographically limited to Newfoundland and Labrador, Canada, which is known for its high prevalence of chronic disease (Statistics Canada, 2023) and unique genetic mutations (Merner et al., 2008; Rahman et al., 2003). As such, the generalizability of our findings to the broader MS population cannot be assured.

## Conclusion

Our longitudinal study in community-dwelling persons living with stable MS demonstrated that CRF does not predict longitudinal changes in behavioral and physiological variables, including hand dexterity, global cognition, and corticospinal excitability. In fact, we found that the strongest predictor of follow-up performance was baseline score and that participants experience heterogenous changes over 2 years, including decline, no change and even improvement. Overall, our study provides important insights into the longitudinal relationship between CRF and MS-related outcomes while highlighting the complexity and variability in MS progression.

## Data Availability

The raw data supporting the conclusions of this article will be made available by the authors, without undue reservation.
