# Supplementary material for: Cardiorespiratory fitness does not predict longitudinal changes in manual dexterity, cognition or corticospinal excitability in multiple sclerosis
Source: Front Aging Neurosci. 2026 Jan 14;17:1628832. doi: 10.3389/fnagi.2025.1628832 (PMC12847388; doi:10.3389/fnagi.2025.1628832)
Supplement: Supplementary file 1 [file Data_Sheet_1.pdf]

Table S1: Spearman's Correlations of Baseline Cardiorespiratory Fitness ($\dot{V}O_2max$) with Baseline Age, Cognition, Hand Dexterity, and TMS measures

Baseline Variable	Spearman Correlation to Baseline $\dot{V}O_2max$	*p* - value
**Age**	-0.223	0.178
**9 Hole Peg Test (seconds)**		
**Dominant**	-0.336	***0.039***
**Non-Dominant**[a]	-0.243	0.142
**Montreal Cognitive Assessment**	0.199	0.230
**Corticospinal Excitability**		
**Active Motor Threshold**[b]	-0.340	***0.040***
**Cortical Silent Period**[c]	-0.396	***0.030***
**Asymmetry Ratios**[d]	-0.123	0.480

All values are Spearman's rho (ρ). We examined correlations between baseline $\dot{V}O_2max$ and demographic, cognitive, motor, and neurophysiological variables. [a]n=35, [b]n=37. [c]n=29, [d]n=28. Bolded and italicized p-values indicate statistical significance (i.e., $p < 0.05$).

Table S2a: Hierarchical Regression for Hand Dexterity (9HPT) and Global Cognition (MoCA) of People with MS Over the Age of 50.

	R^2	F Change	B (SE)	95% CI for B LL	95% CI for B UL	β	*p*
**Hand Dexterity (9HPT)**							
Dominant Hand (n = 19)							
Model 1	0.791	13.278					***<0.001***
Age			-0.205 (0.169)	-0.568	0.158	-0.148	0.247
Sex			0.124 (1.394)	-2.867	3.114	0.011	0.931
Days Between T1 & T2			-0.002 (0.002)	-0.007	0.003	-0.092	0.463
9HPT at T1			1.137 (0.166)	0.782	1.493	0.838	***<0.001***
Model 2	0.792	0.017					***<0.001***
Age			-0.209 (0.179)	-0.595	0.177	-0.148	0.263
Sex			0.245 (1.719)	-3.470	3.959	0.018	0.889
Days Between T1 & T2			-0.002 (0.002)	-0.007	0.003	-0.089	0.493
9HPT at T1			1.127 (0.188)	0.720	1.534	0.757	***<0.001***
$\dot{V}O_2$max *at T1*			-0.015 (0.119)	-0.272	0.241	0.016	0.898
Non-Dominant Hand (n = 19)							
Model 1	0.929	22.167					***<0.001***
Age			-0.021 (0.201)	-0.453	0.411	-0.010	0.920
Sex			1.271 (1.629)	-2.223	4.765	0.077	0.448
Days Between T1 & T2			-0.003 (0.003)	-0.008	0.003	-0.102	0.320
9HPT at T1			1.049 (0.115)	0.802	1.295	0.901	***<0.001***
Model 2	0.931	0.366					***<0.001***
Age			-0.049 (0.211)	-0.506	0.408	-0.024	0.820
Sex			1.870 (1.938)	-2.318	6.058	0.097	0.352
Days Between T1 & T2			-0.003 (0.003)	-0.008	0.003	-0.100	0.341
9HPT at T1			1.021 (0.126)	0.749	1.294	0.819	***<0.001***
$\dot{V}O_2$max *at T1*			-0.082 (0.135)	-0.374	0.210	-0.061	0.555
**Global Cognition (MoCA)** (n=19)							
Model 1	0.781	5.471					***0.007***
Age			-0.017 (0.099)	-0.196	0.229	0.028	0.868
Sex			0.710 (0.795)	-0.995	2.415	0.149	0.387
Days Between T1 & T2			0.002 (0.001)	-0.001	0.004	0.201	0.248
MoCA at T1			0.572 (0.146)	0.259	0.885	0.654	***0.002***
Model 2	0.781	0.000					***0.019***
Age			0.016 (0.104)	-0.209	0.242	-0.137	0.877
Sex			0.719 (0.933)	-1.296	2.734	0.080	0.454
Days Between T1 & T2			0.002 (0.001)	-0.001	0.004	0.136	0.267
MoCA at T1			0.572 (0.152	0.245	0.900	0.648	***0.002***
$\dot{V}O_2$max *at T1*			-0.001 (0.063)	0.137	0.134	-0.054	0.984

Table S2b: Hierarchical Regression for Corticospinal Excitability of People with MS Over the Age of 50.

	R^2	F Change	B (SE)	95% CI for B		β	*p*
				LL	UL		
**Corticospinal Excitability (TMS)**							
AMT (n = 19)							
Model 1	0.764	11.305					***<0.001***
Age			-0.957 (0.302)	-1.605	-0.308	-0.411	***0.007***
Sex			1.955 (2.544)	-3.500	7.411	0.100	0.455
Days Between T1 & T2			0.002 (0.004)	-0.006	0.011	0.085	0.525
AMT at T1			0.662 (0.124)	0.396	0.927	0.695	***<0.001***
Model 2	0.764	0.035					***<0.001***
Age			-0.971 (0.322)	-1.666	-0.275	-0.406	***0.010***
Sex			2.165 (2.867)	-4.027	8.358	0.102	0.463
Days Between T1 & T2			0.002 (0.004)	-0.006	0.011	0.082	0.551
AMT at T1			0.651 (0.140)	0.349	0.954	0.627	***<0.001***
$\dot{V}O_2$max *at T1*			-0.39 (0.209)	-0.492	0.413	-0.025	0.855
CSP (n=16)							
Model 1	0.713	6.821					***0.005***
Age			-3.730 (1.749)	-7.579	0.119	-0.345	0.056
Sex			11.052 (14.156)	-20.104	42.209	0.126	0.452
Days Between T1 & T2			-0.028 (0.021)	-0.076	0.019	-0.214	0.212
CSP at T1			0.748 (0.212)	0.280	1.215	0.569	***0.005***
Model 2	0.743	1.198					***0.009***
Age			-3.831 (1.736)	-7.698	0.037	-0.354	0.052
Sex			17.624 (15.261)	-16.380	51.629	0.185	0.275
Days Between T1 & T2			-0.030 (0.021)	-0.078	0.17	-0.227	*0.187*
CSP at T1			0.724 (0.212)	0.252	1.196	0.548	***0.007***
$\dot{V}O_2$max *at T1*			-1.245 (1.137)	3.779	1.289	-0.175	0.299
Asymmetry Ratios (n = 16)							
Model 1	0.537	3.184					0.058
Age			-0.018 (0.014)	-0.048	0.012	-0.269	0.216
Sex			0.059 (0.112)	-0.188	0.306	0.108	0.610
Days Between T1 & T2			-6.291E-5 (0.00)	0.00	0.00	-0.080	0.705
Asymmetry Ratios at T1			0.599 (0.247)	0.057	1.142	0.499	***0.033***
Model 2	0.540	0.073					0.118
Age			-0.018 (0.015)	-0.50	0.015	-0.260	0.253
Sex			0.039 (0.140)	-0.273	0.350	0.059	0.788
Days Between T1 & T2			-5.995E-5 (0.00)	0.00	0.00	-0.076	0.731
Asymmetry Ratios at T1			0.613 (0.263)	0.028	1.199	0.501	***0.042***
$\dot{V}O_2$max *at T1*			0.003 (0.011)	-0.021	0.026	0.058	0.793

Table S3a: Hierarchical Regression for Hand Dexterity (9HPT) and Global Cognition (MoCA) of People with MS Under the Age of 50.

	R^2	F Change	B (SE)	95% CI for B		β	*p*
				LL	UL		
**Hand Dexterity (9HPT)**							
Dominant Hand (n = 19)							
Model 1	0.734	9.677					***<0.001***
Age			0.031 (0.076)	-0.132	0.193	0.055	0.693
Sex			0.331 (1.634)	-3.173	3.834	0.28	0.843
Days Between T1 & T2			0.005 (0.002)	0.00	0.010	0.290	0.054
9HPT at T1			0.905 (0.148)	0.588	1.223	0.842	***<0.001***
Model 2	0.760	1.387					***<0.001***
Age			-0.005 (0.081)	-0.180	0.169	-0.009	0.950
Sex			0.421 (1.613)	-3.065	3.906	0.035	0.798
Days Between T1 & T2			0.004 (0.003)	-0.002	0.010	0.200	0.165
9HPT at T1			0.848 (0.154)	0.515	1.181	0.748	***<0.001***
$\dot{V}O_2$max *at T1*			-0.105 (0.089)	-0.299	0.088	-0.160	0.260
Non-Dominant Hand (n = 19)							
Model 1	0.817	15.583					***<0.001***
Age			-0.009 (0.065)	-0.149	0.131	-0.15	0.895
Sex			0.432 (1.423)	-2.621	3.484	0.035	0.766
Days Between T1 & T2			0.003 (0.002)	-0.002	0.007	0.157	0.191
9HPT at T1			1.120 (0.144)	0.812	1.428	0.892	***<0.001***
Model 2	0.863	4.405					***<0.001***
Age			-0.055 (0.063)	-0.190	0.080	-0.090	0.397
Sex			0.478 (1.277)	-2.280	3.236	0.038	0.714
Days Between T1 & T2			0.001 (0.002)	-0.003	0.006	0.068	0.517
9HPT at T1			1.074 (0.131)	0.792	1.357	0.844	***<0.001***
$\dot{V}O_2$max *at T1*			-0.142 (0.067)	-0.287	0.004	-0.215	0.056
**Global Cognition (MoCA)** (n= 19)							
Model 1	0.404	2.375					0.102
Age			0.049 (0.034)	-0.025	0.122	0.294	0.176
Sex			0.944 (0.706)	-0.571	2.459	0.276	0.203
Days Between T1 & T2			0.001 (0.001)	-0.001	0.003	0.235	0.273
MoCA at T1			0.458 (0.182)	0.068	0.848	0.520	***0.025***
Model 2	0.416	0.268					0.171
Age			0.044 (0.037)	-0.035	0.123	0.254	0.251
Sex			0.950 (0.725)	-0.617	2.517	0.277	0.213
Days Between T1 & T2			0.001 (0.001)	-0.001	0.003	0.192	0.382
MoCA at T1			0.480 (0.191)	0.066	0.893	0.531	***0.026***
$\dot{V}O_2$max *at T1*			-0.020 (0.039)	-0.104	0.064	-0.110	0.613

Table S3b: Hierarchical Regression for Corticospinal Excitability of People with MS Under the Age of 50.

	R^2	F Change	B (SE)	95% CI for B		β	*p*
				LL	UL		
**Corticospinal Excitability (TMS)**							
AMT (n = 18)							
Model 1	0.563	4.195					***0.021***
Age			0.554 (0.233)	0.051	1.057	0.436	**0.033**
Sex			8.400 (5.540)	-3.568	20.368	0.278	0.153
Days Between T1 & T2			-0.003 (0.006)	-0.016	0.011	-0.081	0.665
AMT at T1			0.301 (0.164)	-0.052	0.655	0.337	0.089
Model 2	0.572	0.236					***0.046***
Age			0.579 (0.245)	0.044	1.113	0.445	***0.036***
Sex			8.191 (5.726)	-4.285	20.667	0.270	0.178
Days Between T1 & T2			-0.001 (0.007)	-0.016	0.014	-0.039	0.842
AMT at T1			0.326 (0.176)	-0.058	0.710	0.349	0.089
$\dot{V}O_2$max *at T1*			0.113 (0.233)	-394	0.620	0.092	0.636
CSP (n=13)							
Model 1	0.711	4.912					***0.027***
Age			0.1272 (0.764)	-0.491	3.034	0.316	0.135
Sex			51.581 (18.358)	9.246	93.915	0.534	0.023
Days Between T1 & T2			-0.017 (0.021)	-0.065	0.030	-0.159	0.427
CSP at T1			0.343 (0.137)	0.027	0.660	0.476	***0.037***
Model 2	0.712	0.036					***0.068***
Age			0.386 (1.015)	-1.015	3.788	0.277	0.214
Sex			51.915 (19.656)	5.437	98.393	0.536	0.033
Days Between T1 & T2			-0.016 (0.023)	-0.070	0.038	-0.141	*0.509*
CSP at T1			0.374 (0.220)	-0.146	0.894	0.345	0.132
$\dot{V}O_2$max *at T1*			0.194 (1.023)	-2.225	2.612	0.038	0.855
Asymmetry Ratios (n = 12)							
Model 1	0.683	3.770					0.061
Age			-0.007 (0.008)	-0.025	0.011	-0.189	0.405
Sex			-0.208 (0.176)	-0.625	0.208	-0.252	0.275
Days Between T1 & T2			-6.2832E-5 (0.00)	-0.001	0.00	-0.077	0.727
Asymmetry Ratios at T1			1.022 (0.276)	0.370	1.674	0.789	***0.008***
Model 2	0.704	0.427					0.117
Age			-0.006 (0.008)	-0.026	0.014	-0.153	0.517
Sex			-0.181 (0.188)	-0.643	0.280	-0.214	0.373
Days Between T1 & T2			1.964E-6 (0.00)	-0.001	0.001	0.002	0.993
Asymmetry Ratios at T1			1.038 (0.289)	0.331	1.744	0.798	***0.011***
$\dot{V}O_2$max *at T1*			0.004 (0.007)	-0.012	0.021	0.145	0.538

Table S4a: Hierarchical Regression for Hand Dexterity (9HPT) and Global Cognition (MoCA) of People with EDSS score below 1

	R^2	F Change	B (SE)	95% CI for B		β	*p*
				LL	UL		
**Hand Dexterity (9HPT)**							
Dominant Hand (n = 12)							
Model 1	0.895	14.876					***0.002***
Age			0.11 (0.060)	-0.131	0.152	0.022	0.864
Sex			1.033 (1.573)	-2.686	4.752	0.81	0.532
Days Between T1 & T2			0.00 (0.003)	-0.007	0.008	0.011	0.930
9HPT at T1			0.918 (0.127	0.617	1.219	0.885	**<*0.001***
Model 2	0.903	0.512					***0.005***
Age			-0.002 (0.064)	-0.159	0.156	-0.003	0.980
Sex			1.145 (1.638)	-2.863	5.153	0.089	0.511
Days Between T1 & T2			0.00 (0.003)	-0.008	0.008	-0.005	0.968
9HPT at T1			0.842 (0.170)	0.427	1.257	0.613	***0.003***
$\dot{V}O_2max$ *at T1*			-0.100 (0.140)	-0.443	0.243	-0.091	0.501
Non-Dominant Hand (n = 12)							
Model 1	0.987	135.719					**<*0.001***
Age			0.033 (0.033)	-0.044	0.111	0.044	0.339
Sex			0.645 (0.859)	-1.386	2.675	0.032	0.477
Days Between T1 & T2			0.001 (0.002)	-0.003	0.004	0.016	0.717
9HPT at T1			1.081 (0.049)	0.965	1.197	0.940	**<*0.001***
Model 2	0.988	0.352					**<*0.001***
Age			0.029 (0.035)	-0.056	0.115	0.038	0.434
Sex			0.698 (0.906)	-1.519	2.916	0.035	0.470
Days Between T1 & T2			0.001 (0.002)	-0.004	0.005	0.015	0.757
9HPT at T1			1.061 (0.062)	0.909	1.212	0.765	**<*0.001***
$\dot{V}O_2max$ *at T1*			-0.043 (0.072)	-0.219	0.134	-0.027	0.574
**Global Cognition (MoCA)** (n=12)							
Model 1	0.718	4.451					***0.042***
Age			0.003 (0.034)	-0.079	0.084	0.016	0.940
Sex			-0.307 (0.865)	-2.353	1.738	-0.071	0.733
Days Between T1 & T2			0.001 (0.002)	-0.003	0.005	0.127	0.546
MoCA at T1			0.695 (0.178)	0.274	1.116	0.784	***0.006***
Model 2	0.723	0.122					***0.098***
Age			0.001 (0.037)	-0.090	0.092	0.006	0.980
Sex			-0.333 (0.928)	-2.604	1.937	-0.077	0.732
Days Between T1 & T2			0.001 (0.002)	-0.003	0.005	0.133	0.559
MoCA at T1			0.708 (0.194)	0.233	1.184	0.783	***0.011***
$\dot{V}O_2max$ *at T1*			-0.022 (0.062)	-0.172	0.129	-0.075	0.739

Table S4b: Hierarchical Regression for Corticospinal excitability of People with MS with EDSS score below 1

	R^2	F Change	B (SE)	95% CI for B		β	*p*
				LL	UL		
**Corticospinal Excitability (TMS)**							
AMT (n = 12)							
Model 1	0.440	1.377					0.333
Age			0.111 (0.235)	-0.446	0.667	0.133	0.653
Sex			-2.036 (6.113)	-16.490	12.419	-0.094	0.749
Days Between T1 & T2			-0.005 (0.013)	-0.35	0.026	-0.101	0.730
AMT at T1			0.441 (0.250)	-0.151	1.033	0.498	0.122
Model 2	0.443	0.029					0.510
Age			0.122 (0.262)	-0.520	0.764	0.142	0.659
Sex			-2.041 (6.587)	-18.158	14.076	-0.094	0.767
Days Between T1 & T2			-0.004 (0.014)	-0.039	0.031	-0.083	0.794
AMT at T1			0.478 (0.348)	-0.373	1.329	0.419	0.218
$\dot{V}O_2$max *at T1*			0.097 (0.574	-1.306	1.501	0.052	0.871
CSP (n = 9)							
Model 1	0.870	6.708					***0.046***
Age			0.989 (0.961)	-1.680	3.658	0.185	0.361
Sex			17.618 (19.677)	-37.015	72.251	0.161	0.421
Days Between T1 & T2			-0.038 (0.036)	-0.138	0.062	-0.191	0.348
CSP at T1			0.903 (0.217)	0.299	1.506	0.748	***0.014***
Model 2	0.879	0.221					***0.127***
Age			1.106 (1.099)	-2.393	4.604	0.202	0.389
Sex			21.613 (23.518)	-53.233	96.459	0.184	0.426
Days Between T1 & T2			-0.041 (0.041)	-0.170	0.088	-0.202	*0.389*
CSP at T1			0.982 (0.295)	0.044	1.919	0.669	***0.045***
$\dot{V}O_2$max *at T1*			0.821 (1.748)	-4.741	6.384	0.094	0.670
Asymmetry Ratios (n = 7)							
Model 1	0.550	0.612					0.697
Age			-0.011 (0.020)	-0.096	0.074	-0.268	0.629
Sex			0.076 (0.298)	-1.206	1.358	0.121	0.822
Days Between T1 & T2			0.00 (0.00)	-0.002	0.002	-0.149	0.782
Asymmetry Ratios at T1			0.495 (1.656)	-1.068	2.058	0.646	0.306
Model 2	0.923	4.801					0.454
Age			-0.036 (0.016)	-0.244	0.171	-0.621	0.268
Sex			0.138 (0.177)	-2.113	2.389	0.216	0.580
Days Between T1 & T2			0.00 (0.00)	-0.004	0.003	-0.358	0.421
Asymmetry Ratios at T1			-0.113 (0.350)	-4.562	4.336	-0.090	0.801
$\dot{V}O_2$max *at T1*			-0.050 (0.023)	-0.340	0.240	-0.610	0.273

Table S5a: Hierarchical Regression for Hand Dexterity (9HPT) and Global Cognition (MoCA) of People with EDSS score of 1 or over

	R^2	F Change	B (SE)	95% CI for B		β	*p*
				LL	UL		
**Hand Dexterity (9HPT)**							
Dominant Hand (n = 26)							
Model 1	0.710	12.829					***<0.001***
Age			-0.006 (0.068)	-0.148	0.136	-0.011	0.929
Sex			0.704 (1.486)	-2.386	3.795	0.056	0.640
Days Between T1 & T2			0.00 (0.002)	-0.004	0.004	0.012	0.919
9HPT at T1			0.964 (0.146)	0.661	1.266	0.779	***<0.001***
Model 2	0.725	1.104					***<0.001***
Age			-0.019 (0.069)	-0.163	0.125	-0.032	0.785
Sex			1.284 (1.582)	-2.016	4.584	0.095	0.426
Days Between T1 & T2			-7.34E-5 (0.002)	-0.004	0.004	-0.004	0.970
9HPT at T1			0.939 (0.147)	0.633	1.246	0.749	***<0.001***
$\dot{V}O_2max$ *at T1*			-0.102 (0.097)	-0.305	0.101	-0.123	0.306
Non-Dominant Hand (n = 26)							
Model 1	0.854	30.623					***<0.001***
Age			-0.030 (0.063)	-0.162	0.101	-0.040	0.636
Sex			2.748 (1.374)	-0.110	5.606	0.167	0.059
Days Between T1 & T2			-0.001 (0.002)	-0.005	0.002	-0.065	0.442
9HPT at T1			0.965 (0.098)	0.760	1.169	0.819	***<0.001***
Model 2	0.867	2.043					***<0.001***
Age			-0.045 (0.062)	-0.175	0.085	-0.059	0.481
Sex			3.438 (1.426)	0.464	6.412	0.196	0.026
Days Between T1 & T2			-0.002 (0.002)	-0.005	0.002	-0.083	0.322
9HPT at T1			0.948 (0.097)	0.746	1.150	0.798	***<0.001***
$\dot{V}O_2max$ *at T1*			-0.125 (0.088)	-0.309	0.058	-0.116	0.168
**Global Cognition (MoCA)** (n=26)							
Model 1	0.464	4.536					***0.008***
Age			-0.043 (0.036)	-0.119	0.033	-0.187	0.255
Sex			0.301 (0.761)	-1.281	1.883	0.063	0.696
Days Between T1 & T2			0.001 (0.001)	-0.001	0.004	0.230	0.165
MoCA at T1			0.514 (0.159)	0.183	0.845	0.516	***0.004***
Model 2	0.464	0.016					***0.020***
Age			-0.042 (0.038)	-0.121	0.036	-0.183	0.276
Sex			0.264 (0.831)	-1.470	1.998	0.052	0.754
Days Between T1 & T2			0.001 (0.001)	-0.001	0.004	0.230	0.176
MoCA at T1			0.510 (0.165)	0.166	0.855	0.506	***0.006***
$\dot{V}O_2max$ *at T1*			0.007 (0.052)	-0.102	0.115	0.021	0.899

Table S5b: Hierarchical Regression for Corticospinal excitability of People with MS with EDSS score of 1 or over

	R^2	F Change	B (SE)	95% CI for B		β	*p*
				LL	UL		
**Corticospinal Excitability (TMS)**							
AMT (n = 26)							
Model 1	0.535	6.049					***0.002***
Age			-0.201 (0.134)	-0.479	0.077	-0.224	0.147
Sex			5.334 (2.824)	0.540	11.207	0.281	0.073
Days Between T1 & T2			-0.001 (0.004)	-0.009	0.007	-0.051	0.737
AMT at T1			0.498 (0.117)	0.256	0.741	0.636	***<0.001***
Model 2	0.536	0.026					***0.006***
Age			-0.197 (0.139)	-0.488	0.093	-0.216	0.172
Sex			5.196 (3.017)	-1.096	11.488	0.262	0.100
Days Between T1 & T2			-0.001 (0.004)	-0.009	0.007	-0.044	0.774
AMT at T1			0.505 (0.126)	0.242	0.769	0.609	***<0.001***
$\dot{V}O_2$max *at T1*			0.033 (0.205)	-0.395	0.461	0.024	0.874
CSP (n=21)							
Model 1	0.515	4.256					***0.016***
Age			-0.003	-1.492	1.485	-0.001	0.996
Sex			18.428	-12.931	49.788	0.217	0.231
Days Between T1 & T2			-0.036	-0.076	0.005	-0.327	0.078
CSP at T1			0.567	0.176	0.957	0.535	***0.007***
Model 2	0.532	0.537					***0.029***
Age			-0.072	-1.603	1.460	-0.018	0.922
Sex			23.076	-11.659	57.811	0.250	0.177
Days Between T1 & T2			-0.038	-0.080	0.004	-0.344	*0.071*
CSP at T1			0.490	0.033	0.947	0.404	***0.037***
$\dot{V}O_2$max *at T1*			-0.844	-3.197	1.610	-0.129	0.475
Asymmetry Ratios (n = 22)							
Model 1	0.702	10.005					***<0.001***
Age			-0.003 (0.004)	-0.011	0.005	-0.100	0.460
Sex			0.027 (0.076)	-0.134	0.188	0.047	0.729
Days Between T1 & T2			-6.39E-5 (0.00)	0.00	0.00	-0.079	0.558
Asymmetry Ratios at T1			0.938 (0.163)	0.593	1.283	0.760	***<0.001***
Model 2	0.720	1.051					***<0.001***
Age			-0.002 (0.004)	-0.011	0.006	-0.069	0.608
Sex			0.001 (0.080)	-0.168	0.171	0.002	0.986
Days Between T1 & T2			-4.73E-5 (0.00)	0.00	0.00	-0.058	0.667
Asymmetry Ratios at T1			0.969 (0.166)	0.617	1.321	0.772	***<0.001***
$\dot{V}O_2$max *at T1*			0.006 (0.005)	-0.006	0.017	0.136	0.321

Note: 9HPT = Nine-Hole Peg Test; MoCA = Montreal Cognitive Assessment; AMT = Active Motor Threshold; CSP = Cortical silent Period; T1 = Baseline; T2 = Follow-up; R^2 = coefficient of determination; F Change = F-statistic for R^2 change, B = unstandardized regression coefficient; SE = standard error; CI = confidence interval; LL = lower limit; UL = upper limit; β = standardized regression coefficient. Bolded and italicized *p*-values indicate statistical significance (i.e., $p < 0.05$).
